# Predicting Responses to Electroconvulsive Therapy in Adolescents with Treatment-Refractory Depression Based on Resting-State fMRI

**DOI:** 10.3390/jcm12103556

**Published:** 2023-05-19

**Authors:** Xiao Li, Jiamei Guo, Xiaolu Chen, Renqiang Yu, Wanjun Chen, Anhai Zheng, Yanjie Yu, Dongdong Zhou, Linqi Dai, Li Kuang

**Affiliations:** 1 Department of Psychiatry, The First Affiliated Hospital of Chongqing Medical University, Chongqing 400016, China; 2 The First Branch, The First Affiliated Hospital of Chongqing Medical University, Chongqing 400015, China; 3 Department of Radiology, The First Affiliated Hospital of Chongqing Medical University, Chongqing 400016, China; 4 Mental Health Center, University-Town Hospital of Chongqing Medical University, Chongqing 401331, China

**Keywords:** depression, functional connectivity, ALFF, prediction, machine learning

## Abstract

Objects: The efficacy of electroconvulsive therapy (ECT) in the treatment of adolescents with treatment-refractory depression is still unsatisfactory, and the individual differences are large. It is not clear which factors are related to the treatment effect. Resting-state fMRI may be a good tool to predict the clinical efficacy of this treatment, and it is helpful to identify the most suitable population for this treatment. Methods: Forty treatment-refractory depression adolescents were treated by ECT and evaluated using HAMD and BSSI scores before and after treatment, and were then divided into a treatment response group and a non-treatment group according to the reduction rate of the HAMD scale. We extracted the ALFF, fALFF, ReHo, and functional connectivity of patients as predicted features after a two-sample *t*-test and LASSO to establish and evaluate a prediction model of ECT in adolescents with treatment-refractory depression. Results: Twenty-seven patients achieved a clinical response; symptoms of depression and suicidal ideation were significantly improved after treatment with ECT, which was reflected in a significant decrease in the scores of HAMD and BSSI (*p* < 0.001). The efficacy was predicted by ALFF, fALFF, ReHo, and whole-brain-based functional connectivity. We found that models built on a subset of features of ALFF in the left insula, fALFF in the left superior parietal gyrus, right superior parietal gyrus, and right angular, and functional connectivity between the left superior frontal gyrus, dorsolateral–right paracentral lobule, right middle frontal gyrus, orbital part–left cuneus, right olfactory cortex–left hippocampus, left insula–left thalamus, and left anterior cingulate gyrus–right hippocampus to have the best predictive performance (AUC > 0.8). Conclusions: The local brain function in the insula, superior parietal gyrus, and angular gyrus as well as characteristic changes in the functional connectivity of cortical–limbic circuits may serve as potential markers for efficacy judgment of ECT and help to provide optimized individual treatment strategies for adolescents with depression and suicidal ideation in the early stages of treatment.

## 1. Introduction

Electroconvulsive therapy (ECT) is considered to be one of the best methods for MDD for all ages [1,2], especially for those with severe treatment-refractory depression. However, the usage of ECT in adolescents is limited and has always been carefully evaluated. In 2004, the AACAP developed practice parameters to assist clinicians considering ECT in treating adolescent patients. In these guidelines, criteria were outlined for selecting patients who might benefit from ECT treatment. Patients must have (1) a diagnosis of severe major depressive disorder, mania, schizoaffective disorder, schizophrenia, catatonia, or neuroleptic malignant syndrome, (2) symptom severity that is persistently disabling or life threatening, and (3) failure of at least two adequate prior treatment regimens when feasible [3]. In addition, because of its side effects and high cost, it is important to investigate which category of patient responds better to ECT; this has significant implications in adolescents with depression. Previous studies have explored how to predict the antidepressant effects of ECT; a structural MRI study focusing on ECT found that it caused an increase in hippocampal gray matter volume (GMV) and can predict the effect of ECT [4].

The efficacy of ECT has been found to correlate with an increased fractional amplitude of low-frequency fluctuation (fALFF) in regions such as the prefrontal cortex, insula, and hippocampus, as well as increased GMV in regions such as the anterior cingulate gyrus, insula, thalamus, caudate nucleus, and hippocampus [5]. These studies based on longitudinal data have explored longitudinal alterations in the brain induced by ECT, providing new insights into the therapeutic mechanisms of ECT. However, few studies have distinguished the differences in the patterns of response and non-response to ECT in adolescents with treatment-refractory depression based on resting state fMRI (rs-fMRI). Therefore, it is important to investigate the brain function differences between responders and non-responders to ECT to help provide meaningful biomarkers to identify responders. Previous studies have found that functional brain imaging metrics can be used to predict treatment outcomes in patients with depression. In a meta-analysis, functional connectivity (FC) in the DMN of depressed patients was found to predict the efficacy of ECT [6]. Another study focused on FC as a predictor to explore its predictive effect in depressed patients after ECT and found that FC within the DMN as well as FC between the DMN and CEN could be used as predictors of response to ECT in depressed patients [7].

With the development of artificial intelligence (AI), machine learning techniques have also started to be frequently used in the diagnosis and risk prediction of depression. Cui et al. [8] developed an fMRI-based method for diagnosing schizophrenia by machine learning techniques with an accuracy of 87%. Methods such as the support vector model (SVM) and least absolute shrinkage and selection operator (LASSO) can build models based on existing data and then achieve prediction for new datasets. Previous studies have found that SVM as well as LASSO can achieve more than 90% accuracy in diagnosing MDD based on fMRI features [9]. Therefore, we consider that fMRI-based neurological features may be potential indicators related to the response to ECT in adolescents with treatment-refractory depression.

The aim of the study is to investigate the pattern differences at baseline in adolescents with treatment-refractory depression with different responses to ECT. Predictive models for the responding and non-responding groups were constructed using analyses based on ALFF, fALFF, ReHo, and FC. We used a logistic regression model (LRM) as well as SVM for prediction, and brain imaging at baseline of adolescents showing a response to ECT may help to expand our understanding of brain function predictors of the response to ECT.

## 2. Materials and Methods

### 2.1. Participants

Forty adolescents with treatment-refractory depression between 12 and 17 years of age were enrolled. The diagnosis was confirmed by two psychiatrists using the Mini International Neuropsychiatric Interview for Children and Adolescents (MINI-KID). The inclusion criteria were as follows: (1) the Hamilton Depression Rating Scale (HAMD 17) *≥* 17; (2) with a diagnosis of treatment refractory depression and no history of ECT treatment; and (3) patients with severe suicidal ideation (Beck Scale for Suicide Ideation, BSSI *≥* 11) in the past week. Participants were excluded if they: (1) had a neurological or serious physical condition, any history of alcohol or drug abuse, any other somatic diseases, or morphological anomalies of the brain; (2) had any surgically placed electronic or metal materials that might interfere with fMRI assessment; or (3) had head motion exceeding 2.5 mm in translation or 2.5° in rotation. The clinical scale was assessed using HAMD and BSSI, response rate = (pre-treatment HAMD − post-treatment HAMD)/pre-treatment HAMD; if the response rate was ≥50%, they were considered to be responders, whilst if the response rate was <50%, they were considered to be non-responders. The study protocol was approved by the Human Research and Ethics Committee of the First Affiliated Hospital of Chongqing Medical University (no. 2017-157). Written informed consent was obtained from all adolescents and their parents. We registered the study in the Chinese Clinical Trial Registry, the ID is ChiCTR2200064527.

### 2.2. Electroconvulsive Therapy

Modified bitemporal ECT was delivered by Thymatron DGx (Somatics, LLC, Lake Bluff, IL, USA) at the First Affiliated Hospital of Chongqing Medical University. The first three sessions of ECT took place on continuous days; the remaining were performed every 2 days, with a break on weekends; after eight sessions, the ECT was completed. The first energy for ECT was 5% for all patients. The stimulation energy was adjusted based on the seizure time. The energy was increased by 5% in the subsequent treatment if the seizure time was <25 s. Anesthesia was induced with succinylcholine (0.5–1 mg/kg) and propofol (1.5–2 mg/kg).

### 2.3. Image Data Acquisition

MR images were obtained using a 3T GE Signa HDxt scanner (General Electric Healthcare, Chicago, IL, USA) with an eight-channel head coil. Participants were instructed to relax with their eyes closed, stay awake, and avoid thinking as much as possible. None of the patients reported falling asleep during the scan. Foam pads and earplugs were used to fix their heads to minimize head motion and reduce machine noise, respectively. The echo-planar imaging pulse sequence parameters were as follows: repetition time (TR) = 2000 ms; echo time (TE) = 40 ms; field of view (FOV) = 240 × 240 mm^2^; matrix = 64 × 64; flip angle = 90°; slice number = 33; slice thickness/gap = 4.0/0 mm; scanner time = 8 min; and 240 volumes. Three-dimensional T1-weighted MR images were used for rs-fMRI co-registration (TR = 24 ms; TE = 9 ms; FOV = 240 × 240 mm^2^; matrix = 256 × 256; flip angle = 90°; and slice thickness/gap = 1.0/0 mm).

### 2.4. Rs-fMRI Preprocessing and Feature Extraction

The data were preprocessed using DPARSF software. The specific preprocessing process is as follows. (1) Data format conversion: convert DICOM format to NIFTI format. (2) Remove first time point: remove the initial 10 time points. (3) Slice timing: correct each layer of scanned data to one time point. (4) Realignment: covariate processing of white matter signals, cerebrospinal fluid signals, and head movement parameters (translation ≤ 2.5 mm, rotation ≤ 2.5°) using the Fristion-24 model. (5) Spatial normalization: because of the differences in subject head size, we need to perform spatial normalization, as follows. DARTEL alignment is used to align the brain fMRI images to the standard brain template, the standardized images need to be resampled, and the spatial resolution of the resampled functional images is 3 mm × 3 mm × 3 mm. (6) Spatial smoothing: the purpose is to reduce the impact of spatial noise on the data and reduce the differences in brain structure between subjects. (7) Detrending: during data acquisition, the noise of the machine will gradually increase, and this process may make our data change accordingly, so we need to complete the detrending to remove this effect. (8) Low-pass filter (0.01–0.10 Hz): the purpose of this is to reduce the impact of physiological noise such as the subject’s breathing and heartbeat on the acquired data. The next step is to calculate the whole-brain ALFF, fALFF, and ReHo for all patients; feature extraction based on the AAL template extracts the average ALFF, fALFF, and ReHo for the 90 brain regions of the patient group and FC for each brain region based on the whole brain as features.

### 2.5. Model Building and Validation

Based on the dataset of 40 patients, 75% of the data was randomly selected as the training set, and the remaining 25% was used as the validation set. Based on the training set, feature selection was performed as follows: (1) a two-sample *t*-test was performed for each feature, and features with *p* < 0.05 were screened; (2) the optimal subset of features was selected using LASSO. In this method, the parameter λ controls the balance between data fit and sparsity, which is a parameter determined by using 10-fold cross-validation. Based on the selected features, LRM and SVM are constructed. SVM are a class of generalized linear classifiers that perform the binary classification of data in a supervised learning manner, where the decision boundary is a hyperplane of maximum margins for the learned samples, which can be reduced to a problem of solving convex quadratic programming. For the model built from the training set, the performance of the model is evaluated using a test set. The evaluation metrics include the accuracy (ACC), sensitivity (SEN), specificity (SPEC), positive prediction rate (PPV), negative prediction rate (NPV), area under the ROC curve (AUC), and discriminant performance (AUC: 0.9–1.0 = excellent; 0.8–0.9 = good; 0.7–0.8 = moderate; 0.6–0.7 = poor; 0.5–0.6 = failure). The following R packages (The University of Auckland, Auckland, New Zealand) were used for pattern classification analysis: (http://www.R-project.org, version: 3.6.1).

## 3. Results

### 3.1. Clinical Characteristics

A total of 40 adolescents with treatment-refractory depression enrolled in the study completed ECT. Of these, 27 (67.5%) patients achieved a clinical response (HAMD rate ≥ 50%). The other 13 (22.5%) did not achieve a clinical response (HAMD reduction rate < 50%), and there were no significant differences in age, gender, education, disease duration, HAMD, or BSSI scores between the two groups at baseline. After ECT, both groups showed a reduction in depressive and suicidal ideation scores (Table 1).

### 3.2. Feature Selection

**Figure 1:**
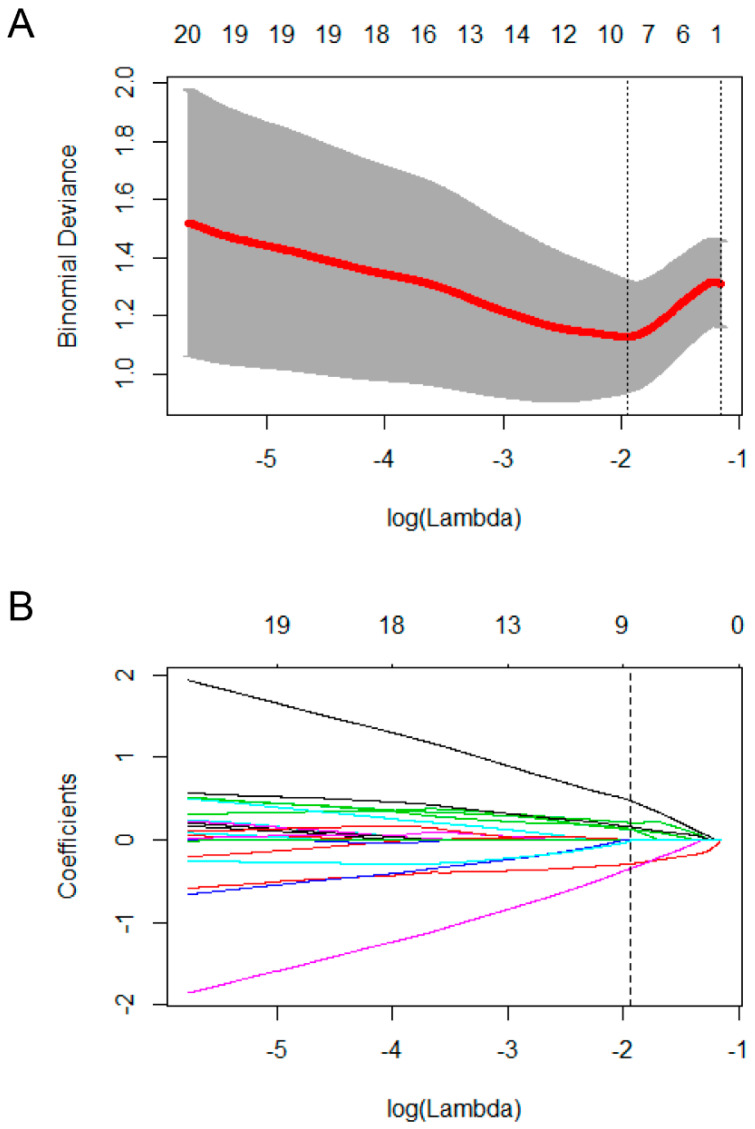
(**A**) Nine features corresponding to the binomial deviance are selected by ten–fold cross–validation; (**B**) coefficients of features.

Figure 1A,B show the results of LASSO feature selection, and Table 2 lists the feature information selected by LASSO. Nine features were finally selected: (1) ALFF of the left insula (INS.L); (2) fALFF of the left superior parietal gyrus (SPG.L); (3) fALFF of the right superior parietal gyrus (SPG.R); (4) fALFF of the right angular gyrus (ANG.R); (5) FC of left superior frontal gyrus, dorsolateral–right paracentral lobule (SFGdor.L–PCL.R); (6) FC of orbitofrontal middle gyrus–left cuneus (ORBmid.R–CUN.L); (7) FC of right olfactory cortex–left hippocampus (OLF.R–HIP.L); (8) FC of left insula–left thalamus (INS.L–THA.L); and (9) FC of the left anterior cingulate and paracingulate gyri–right hippocampus (ACG.L–HIP.R).

### 3.3. Validation Performance of LRM and SVM

**Figure 2:**
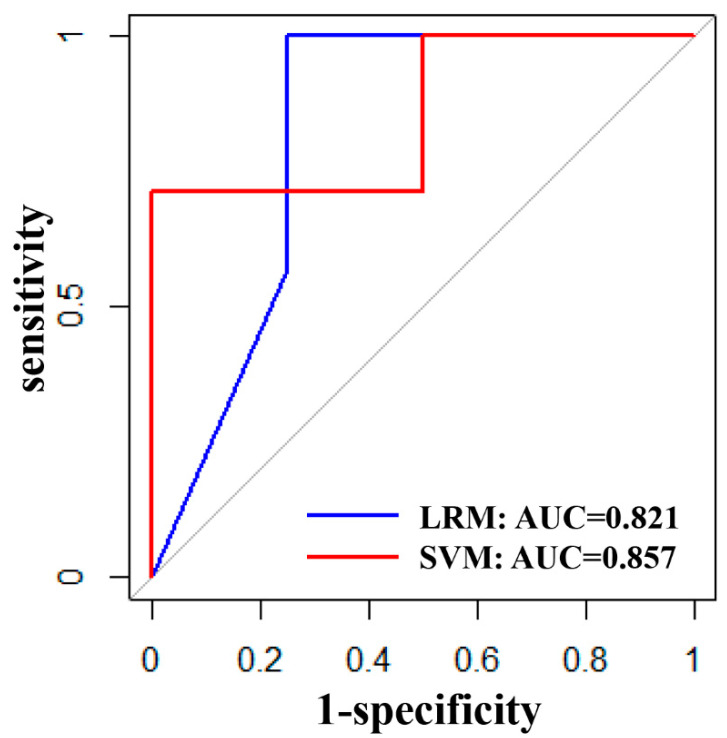
Sensitivity and specificity of LRM and SVM; blue indicates LRM with AUC = 0.821 and red indicates SVM with AUC = 0.857.

The performance of the LRM and SVM built based on the optimal subset of features selected for the training set in the validation set is shown in Table 3, with close performance, both showing good efficacy in prediction performance (AUC > 0.8), sensitivity and specificity, and ROC curves as shown in Figure 2.

**Figure 3:**
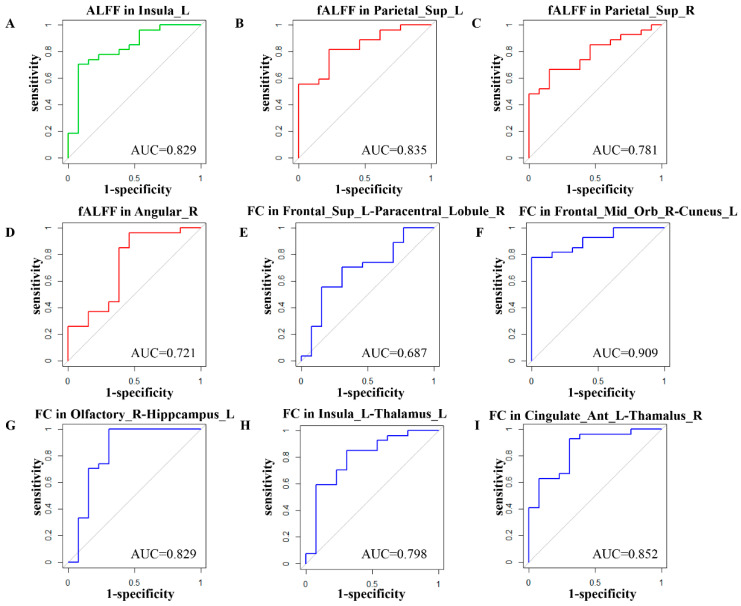
Prediction performance of nine brain regions, (**A**): AUC = 0.829 for ALFF of the INS.L; (**B**): AUC = 0.835 for fALFF of the SPG.L; (**C**): AUC = 0.781 for fALFF of the SPG.R; (**D**): AUC = 0.721 for fALFF of the ANG.R; (**E**): AUC = 0.687 for FC of SFGdor.L–PCL.R; (**F**): AUC = 0.909 for FC of ORBmid.R–CUN.L; (**G**): AUC = 0.829 for FC of OLF.R–HIP.L; (**H**): AUC = 0.798 for FC of INS.L–THA.L; (**I**): AUC = 0.852 for FC of ACG.L–HIP.R.

The predictive performance of the brain function of nine brain regions for efficacy was further evaluated based on the full data, which showed that the AUC of the patient group at baseline was 0.829 for ALFF of the INS.L, 0.835 for fALFF of the SPG.L, 0.781 for fALFF of the SPG.R, 0.721 for fALFF of the ANG.R, 0.687 for FC of SFGdor.L–PCL.R, 0.909 for FC of ORBmid.R–CUN.L, 0.829 for FC of OLF.R–HIP.L, 0.798 for FC of INS.L–THA.L, and 0.852 for FC of ACG.L–HIP.R; the ROC curves are shown in Figure 3.

## 4. Discussion

This study investigated the possible predictive ability of brain functional imaging to predict whether adolescents with treatment-refractory depression respond to ECT. Our results showed that the accuracy of LRM is 81.8% and accuracy of SVM is 81.8%. We found that the ALFF of INS.L, the fALFF of SPG.L, SPG.R, ANG.R, and the FC of SFGdor.L–PCL.R, ORBmid.R–CUN.L, OLF.R–HIP.L, INS.L–THA.L, and ACG.L–HIP.R may predict the response to ECT in adolescents with depression. The present results suggest that local brain function and FC may serve as potential predictors of response to ECT in depressed adolescent patients.

Both the dorsolateral superior frontal gyrus and the orbitofrontal middle gyrus are important regions of the prefrontal cortex, and the dorsolateral prefrontal cortex is thought to have a relatively important role in working memory and emotion regulation [10,11], while the orbitofrontal cortex is thought to be involved in activities such as emotional cognition as well as decision processing [12,13]. Previous studies have focused on the relationship between changes in the prefrontal cortex and depression, such as studies based on morphological changes that showed a decrease in prefrontal volume in depressed patients. Bora et al. found that patients not treated with antidepressants had significantly smaller prefrontal volumes than those treated with antidepressants [14]. They also found a reduction in the volume of the dorsolateral prefrontal cortex in depressed patients [15].

The relationship between the prefrontal cortex and depression has also been explored in more studies in rs-fMRI-based studies, such as Zhang et al. [16], who found that the FC of the right prefrontal cortex to the amygdala was significantly increased in depressed patients compared with normal controls. Our study found that the FC of the SFGdor.L–PCL.R and ORBmid.R–CUN.L could be used to predict the effect of ECT in depressed adolescents. A review also focused on the prediction of rs-fMRI for ECT found that the FC of the prefrontal cortex correlated with the response of ECT, wherein the FC of the middle frontal gyrus, superior frontal gyrus, and orbit could predict the effect of ECT in depressed patients [17]. In another study, Chen et al. [18] found that the FC in the prefrontal areas could predict the effect of repetitive transcranial magnetic stimulation (rTMS) on depression, and the regions with the most predictive value were the dorsolateral prefrontal, ventral lateral prefrontal, orbitofrontal, and medial prefrontal. This is similar to our findings and also suggests that the function of brain regions is more closely related to mood regulation, such as the dorsolateral prefrontal and orbitofrontal, which are more predictive of treatment response, no matter what the intervention is.

We found that an abnormal FC in subcortical areas may predict the response to ECT in adolescents with treatment-refractory depression, such as INS and ACG, and also found that both the local brain function and FC in INS.L were predictive of the response to ECT. INS is an important component of the limbic system, which is thought to represent cognitive, emotional, and visual capacities. Studies have also demonstrated that there is a reduction in the FC of INS with other brain regions in depressive patients, such as the anterior cingulate cortex, which can indicate the severity of depressive symptoms [19]. Bouckaert et al. found that the gray matter volume and cortical thickness of INS increased after ECT in depressed patients [19]. Wang et al. found that the FC of INS with the angular gyrus and dorsolateral prefrontal cortex increased in depressed patients after ECT, while the functional activity of the INS.R may be a marker of ECT response in depressed patients [20]. There is a correlation between INS and ECT response in depressed patients [19], suggesting that it may be a useful predictive biomarker; fMRI studies also showed that the resting-state network connectivity of the INS can predict ECT efficacy [21]. In addition, we analyzed that the FC of ACG.L–HIP.R predicted the therapeutic effect of ECT. The cingulate gyrus belongs to the limbic system, and the ACG is a significant region of the cingulate gyrus that is closely related to emotion, cognition, and reward [22]. Studies are common on the relationship between ACG and depression, such as Lai [23], who found a reduction in cingulate gyrus volume in depressed patients compared with healthy controls. In terms of brain metabolism studies, Auer et al. [24] presented data on abnormalities in glucose metabolism in the ACG in depressed patients, and in FC studies, abnormalities in this region were also noticed in depressed patients [25]. HIP is generally considered to be related to memory [26], and there are more inconsistent results in previous studies on HIP and depression, such as some studies that showed reduced HIP volume in depressed patients [27]; however, Vakili et al. [28] found no significant difference in HIP volume in depressed patients compared with healthy controls, but for depressed patients who were treated with ECT, the finding of increased HIP volume is generally consistent [29], and it has been suggested that HIP volume at baseline may predict the effect of ECT [4]. We considered that the FC of the ACG.L–HIP.R could predict the efficacy of ECT in depressed adolescents, and a recent fMRI study focusing on assessing ECT for depression showed that ECT may change the FC based on ACG or HIP as ROIs, and that HIP-based FC could predict the treatment effect of ECT [17]. This is similar to our findings.

The parietal cortex, which plays a crucial role in the process of focusing attention, mainly includes the postcentral gyrus, superior parietal gyrus, and inferior parietal gyrus. The parietal lobe of the brain is composed primarily of the sensory and cortical components that monitor the response of various body parts to external stimuli. In addition, the parietal lobe is also involved in working memory and attentional capacity [30]. In our study, we concluded that fALFF in the bilateral superior parietal gyrus predicted the therapeutic effect of ECT. Previous studies focusing on psychiatric disorders with suicidal behavior have shown a marked reduction in gray matter volume in the left superior parietal gyrus in patients compared with healthy controls [31]. Several studies have indicated that metabolic deficits in the parietal lobe are altered in patients with depression with suicidal ideation; Heeringen et al. [32] found that depressed patients with suicidal ideation had reduced metabolic activity in the right inferior parietal gyrus, and that reduced brain activity correlated with suicidal ideation.

Methodologically, this study used both LRM and SVM, which were applied to demonstrate the validity of the results. Validation algorithms were used to verify the reproducibility and reliability. Machine learning studies will have an impact on precision medicine. The development of radiomics has created an ideal environment for the application of brain imaging in psychiatry, where psychiatric disorders do not yet appear as “lesions” in conventional brain imaging, but where markers and features predictive of mental disorders can still be obtained. We gathered functional brain activity features from functional brain imaging, and stepwise analysis can facilitate our findings to change clinical practice, which can contribute to the dissemination of potential markers.

There are still some limitations; first, we used medications in our study, so we could not exclude the impact of medications. Second, the prediction of this study mainly includes the brain function feature, such as ALFF, fALFF and functional connectivity. This result may not be reliable enough in other samples, so more indicators can be included in future research, such as biochemistry, gene, EEG, cognition and others, which may enhance the predictive effect of treatment response to depression. Third, because our sample size is small and has not been validated in an independent sample, our results should be treated with caution that may not apply to other independent samples. Due to the small amount of data, this study only preliminarily proves the prediction performance of functional characteristics. The sample should be further increased, especially by incorporating a multi-center sample, to establish a more reliable and better performance model. Finally, we did not observe the long-term effects of ECT for adolescents, so further research is needed.

In summary, altered brain function in adolescents with treatment-refractory depression may be an important cause of symptom onset, which also suggests that brain function may play a predictive role for the effect of ECT in depressive adolescents. This study explores the predictive power of local brain function and functional connectivity on the efficacy of ECT in adolescent with treatment-refractory depression, and the results show high accuracy. It also evokes that brain regions such as the superior frontal gyrus, superior parietal gyrus, cingulate cortex, hippocampus, thalamus, and insula have the potential to predict the response to ECT.

## Figures and Tables

**Table 1:** Clinical demographics of response group and non-response group.

Feature	Responder (*n* = 27)	Non-Responder (*n* = 13)	*p*
Male (M/F)	8/19	5/8	0.635 ^#^
Age (y)	16.36 (1.53)	15.04 (1.35)	0.36 *
Education (y)	9.95 (1.73)	8.35 (1.64)	0.165 *
Duration of illness (y)	1.3 (0.9)	1.2 (1.1)	0.376 *
HAMD score pre-ECT	29.45 (5.72)	30.21 (6.03)	0.662 *
HAMD score post-ECT	11.36 (5.43)	18.32 (4.53)	<0.001 ^+^
BSSI score pre-ECT	23.88 (6.34)	22.94 (5.63)	0.532 *
BSSI score post-ECT	5.56 (3.72)	14.32 (4.86)	<0.001 ^+^
Number of ECT	8.3 (2.0)	8.1 (2.9)	0.67 *
Antidepressants dose (mg/d) ^a^	49.3 (7.9)	51.8 (6.4)	0.54 *

^#^: chi-square test, *: two-sample *t*-test, ^+^: paired *t*-test. ECT: electroconvulsive therapy; HAMD: Hamilton Depression Rating Scale; BSSI: Beck Scale for Suicide Ideation; ^a^ Fluoxetine equivalents based on defined daily doses method.

**Table 2:** Coefficients and prediction performance evaluation of LASSO-selected features.

Number	Brain Region	Feature	Coefficient
1	INS.L	ALFF	0.289
2	SPG.L	fALFF	0.209
3	SPG.R	fALFF	0.136
4	ANG.R	fALFF	0.001
5	SFGdor.L–PCL.R	FC	−0.007
6	ORBmid.R–CUN.L	FC	0.478
7	OLF.R–HIP.L	FC	0.127
8	INS.L–THA.L	FC	−0.348
9	ACG.L–HIP.R	FC	0.161

**Table 3:** Performance of LRM and SVM on the validation set.

	ACC	SPENC	SPEC	PPV	NPV	AUC
LRM	81.8%	100%	50%	77.8%	100%	0.821
SVM	81.8%	100%	50%	77.8%	100%	0.857

ACC: accuracy; SENS: sensitivity; SPEC: specificity; PPV: positive predictive value; NPV: negative predictive value; AUC: area under the receiver operating characteristic curve; LRM: logistic regression model; SVM: support vector model.

## Data Availability

The raw data supporting the conclusions of this article will be made available by the authors, without undue reservation.
